# Modelling the public health impact of male circumcision for HIV prevention in high prevalence areas in Africa

**DOI:** 10.1186/1471-2334-7-16

**Published:** 2007-03-13

**Authors:** Nico JD Nagelkerke, Stephen Moses, Sake J de Vlas, Robert C Bailey

**Affiliations:** 1 Department of Community Medicine, United Arab Emirates University, United Arab Emirates; 2 Departments of Medical Microbiology, Medicine and Community Health Sciences, University of Manitoba, Winnipeg, Canada; 3 Department of Public Health, Erasmus MC, University Medical Center Rotterdam, Rotterdam, The Netherlands; 4 Division of Epidemiology, University of Illinois at Chicago, Chicago, USA

## Abstract

**Background:**

Recent clinical trials in Africa, in combination with several observational epidemiological studies, have provided evidence that male circumcision can reduce HIV female-to-male transmission risk by 60% or more. However, the public health impact of large-scale male circumcision programs for HIV prevention is unclear.

**Methods:**

Two mathematical models were examined to explore this issue: a random mixing model and a compartmental model that distinguishes risk groups associated with sex work. In the compartmental model, two scenarios were developed, one calculating HIV transmission and prevalence in a context similar to the country of Botswana, and one similar to Nyanza Province, in western Kenya.

**Results:**

In both models, male circumcision programs resulted in large and sustained declines in HIV prevalence over time among both men and women. Men benefited somewhat more than women, but prevalence among women was also reduced substantially. With 80% male circumcision uptake, the reductions in prevalence ranged from 45% to 67% in the two "countries", and with 50% uptake, from 25% to 41%. It would take over a decade for the intervention to reach its full effect.

**Conclusion:**

Large-scale uptake of male circumcision services in African countries with high HIV prevalence, and where male circumcision is not now routinely practised, could lead to substantial reductions in HIV transmission and prevalence over time among both men and women.

## Background

UNAIDS estimates that 38.6 million people globally are currently living with HIV, and that in 2005, 4.1 million new HIV infections occurred [1]. Although the life expectancy of people living with HIV has increased substantially with the introduction of anti-retroviral therapy (ART), it is still uncertain whether ART will have a major impact in reducing the spread of HIV[2]. More than two decades after the discovery of HIV, the range of public health measures to control sexual transmission is still limited. The ideal intervention, of course, would be a highly effective vaccine that offers long-lasting protection, but as yet the search for an effective vaccine has proven elusive, and it is by no means certain that one will become available in the foreseeable future.

Until recently, the growing evidence for a protective effect of male circumcision (MC) against HIV infection has received comparatively little attention from health authorities, despite a dearth of proven effective alternative preventive interventions. The first reports that male circumcision may protect against female-to-male HIV transmission appeared very early during the HIV epidemic[3]. Since then, the evidence for such protection has been accumulating. Associations have been found both ecologically and epidemiologically[4-8], and recently also experimentally[9]. In a randomized clinical trial carried out in the Orange Farm community of South Africa, 20 out of 1545 circumcised men (incidence rate 0.85/100 person years) became infected, compared to 49 out of 1582 men in the control group of delayed circumcision (incidence rate 2.1/100 person years). With an estimated relative risk of infection of 0.40 when analyzed on an intention-to-treat basis, MC had a very high protective effect. This protection changed only marginally to a relative risk of 0.39 when taking into account behavioural factors, including possible increases in risk taking (behavioural disinhibition or risk compensation[10]) after circumcision. In fact, the relative risk estimate was even lower, 0.24, when the data were analyzed on a per-protocol basis. Similar trials have been undertaken in Nyanza Province (Kisumu), in Kenya[11-13] and Rakai, Uganda[14,15]. Although results have not yet been fully published, the Kenyan and Ugandan trials were stopped in December 2006, as the Data Safety and Monitoring Board determined that the protective effect of MC was sufficiently strong that continued enrolment was unethical, and it was necessary to offer circumcision to the control groups [16,17]. In Kenya, it was found that only 22 of the 1,393 circumcised men enrolled in the study were infected with HIV, compared to 47 of the 1,391 men who had yet to be circumcised, giving an estimated protective effect of 53%. In Uganda, 4,996 HIV-negative, uncircumcised men were enrolled, equally between circumcision and control groups. With 43 infected among the uncircumcised men and 22 among the circumcised men, it found an almost identical estimated protective effect of 48%. Although the protective effects of circumcision were slightly less than those found in the Orange Farm trial, the results of these more recent trials are clearly consistent with the South African trial, making the case for a protective effect of MC very strong. Together they appear to constitute overwhelming evidence that male circumcision can reduce female-to-male HIV transmission by 45–65% in sub-Saharan Africa. Another Rakai trial will also analyze the impact of male circumcision on male-to-female HIV transmission. Some evidence that MC reduces male-to-female transmission, especially when the male has low viral load, was provided by a study in Rakai on discordant couples[18,19].

While the epidemiological evidence and the clinical trials provide cogent evidence for the impact of MC on HIV transmission, it is still unclear what the public health impact would be of male circumcision introduced on a large scale, and whether it would be sufficiently effective and cost-effective to justify its introduction as a widespread preventive measure[20]. Unlike prevention efforts with non-communicable diseases, this impact is by no means obvious. First, in highly sexually active men, a lower per-contact risk of transmission may only delay but not prevent infection, thereby reducing the effect of the intervention. Second, protecting men from getting infected may have effects similar to herd immunity. As preventing men from becoming infected clearly also protects their subsequent sex partners, the effects of large scale MC may be larger than expected on the basis of simply examining female-to-male transmission relative risks. Third, it is not known how long it would take to observe a substantial drop in HIV incidence and prevalence, or how long it would take until a new equilibrium situation would be reached. Finally, it is not well understood how MC and other interventions, e.g. female sex worker-focused preventive interventions, would interact. The "gold-standard" for answering these questions, and for assessing the long term effects of large scale introduction of MC, would be a community randomized trial, i.e. to randomize entire communities instead of individuals. Such a trial, however, would be extremely costly and impractical, requiring large sample sizes and follow-up of likely over a decade.

There are two alternatives to such a trial. The first is to resort to ecological data, i.e. comparisons among regions or countries. Although clearly not randomized, and potentially subject to ecological fallacy (e.g. confounding by religious practices, tribal cultural differences, etc.), the results of the South African trial appear to justify a causal interpretation of the observed ecological differences in HIV epidemiology between MC and non-MC regions. These ecological differences have been well documented, and were among the first observations suggesting a protective effect of MC. Typically, countries or regions in Africa, such as West Africa, but also parts of East Africa (for example much of Kenya and Ethiopia) where male MC is widely practised, appear to have much lower HIV prevalence than parts of Africa where MC is rare[4,6], and adult HIV prevalence rarely exceeds 10%. On the other hand in non-MC areas, mostly in the zone ranging from the African Great Lakes Region to KwaZulu Natal in South Africa, adult HIV prevalence in many places exceeds 20%.

The second alternative is to use modelling, in which sets of mathematical equations or (stochastic) computer simulations are used to mimic the HIV epidemic. To model the impact of MC on the HIV epidemic, different approaches can be taken. One approach was published recently[21]. However, it only explores full MC coverage, i.e. a scenario where all men are circumcised before becoming sexual active. This level of circumcision coverage is unlikely to be achieved by even the best of circumcision programs. Also, some men who ultimately accept MC may delay it until after they become sexually active, and therefore be at higher risk for some time. Furthermore, while the authors recognize that ignoring heterogeneity in sexual activity would lead to a predicted steady state prevalence of HIV that is much higher than is generally observed, they incorporated this heterogeneity into their model only indirectly. Instead of deriving the epidemiology of HIV from assumed heterogeneity in sexual behaviour, they postulate an ad-hoc exponential relationship between sexual contact rates and HIV prevalence. While this may be adequate as a statistical tool to fit observed South African data, it is unclear whether this is satisfactory for predicting events under different circumstances such as the effects of an MC program in a wide range of African countries, possibly differing substantially from South Africa. Here, we will present two other approaches: 1) a simple mathematical model assuming random (proportionate) mixing, and postulating explicitly assumptions about heterogeneity in sexual behaviour, to identify what the effect of MC on the epidemiology of HIV could be under this assumption; and 2) to make more specific predictions, a compartmental deterministic model, originally developed to explore the effects of other HIV interventions, such as vaccines, ART, or sex-worker focused interventions[22]. We introduced male circumcision into these models and then calculated HIV transmission over time. We used two different models, instead of only one, as a reciprocal sensitivity analysis: only findings consistent between our two models, each of which makes different simplifying assumption, are probably robust with respect to model misspecification. Findings made in only one model would seem to be crucially dependent on one of the assumptions of that model, and violation of that assumption may invalidate conclusions.

## Methods

We focused on the impact of MC on two aspects of the HIV epidemic, *viz*. its basic reproduction number, R_0_, and the gender specific HIV prevalence. R_0 _is the average number of secondary infections generated by a typical HIV case in a "naïve" population, i.e. when the infection has just been introduced. In both the random mixing and the compartmental model, we used as the baseline relative risk the intention-to-treat estimate found in the Orange Farm study, *viz*. 0.40, corresponding to 60% protection.

### Random mixing model

The simplest, and one of the first and most widely used mathematical models for the HIV epidemic, because of its relative mathematical tractability, is the "Anderson-May" model[23-25]. It assumes random mixing, i.e. individuals randomly choose from among the number of partnerships that are "on offer", at a (per individual) constant rate c_i_. The population distribution of this rate can be continuous (unlike compartmental models), and realistically has a long "tail", representing the fact that few people have high, but most people have low, rates of partner change. Heterosexual contact is assumed, and partners from the opposite sex are selected proportional to their (i.e. the partners') rate of partner change. Thus the rate of forming a partnership between two specific individuals is proportional to the product of the rate of partnership of the two individuals. The model further assumes homogeneous infectivity during the duration D (D_m _in males, D_f _in females) of HIV infection, and a fixed risk of transmission per partnership, *viz*. β_mf_, the risk of an infected male transmitting HIV to an uninfected female partner during their entire partnership, and similarly β_fm_, the female-to-male risk.

Let x_i _denote either β_fm_c_i_D_m _or β_mf_c_i_D_f_, the "scaled mixing rates" of male and female individuals (indexed by i). Let N^m^(x) denote the probability density of the distribution of x among men, and N^f^(x) the same among females. Let I^m^(x)dx and I^f^(x)dx denote the proportion of the population with scaled mixing rate between x and x+dx *and *that is HIV infected (and hence assumed to be infective as well). Further, let N^m ^_1_, and N^m ^_2 _denote for males the expectation of x and x^2 ^respectively with respect to the distribution N^m^(x), and N^f ^_1 _and N^f ^_2 _the same for females. If all males and all females had the same scaled mixing rate N^m ^_1 _and N^f ^_1_, respectively, then the male and female HIV prevalence could be simply calculated from the fact that pre-emption (When the prevalence of HIV is high, infectious individuals often choose partners who are already infected themselves, and cannot therefore become infected. This mechanism is called pre-emption [26]) is the only density dependent mechanism of HIV transmission. As all men and women would have the same rate of partner change, the prevalence π^m ^and π^f ^among random male and female partners would be the same as among the population as a whole, and we would have

π^m ^= N^m ^_1_π^f^(1-π^m^)

and similarly

π^f ^= N^f ^_1_π^m^(1-π^f^)

yielding both π^m ^and π^f ^as a function of the male and female scaled mixing rates, *viz*.

π^m ^= (N^m ^_1 _N^f ^_1 _- 1)/{(N^m ^_1_+1) N^f ^_1_}

and

π^f ^= (N^m ^_1 _N^f ^_1 _- 1)/{(N^f ^_1_+1) N^m ^_1_}

Under this homogeneity assumption (which is highly unrealistic), R_0 _^2 ^= 1/{(1-π^m^)(1-π^f^)} = N^m ^_1 _N^f^_1_, i.e. the product of the two scaled mixing rates. In reality, of course, scaled mixing rates are highly heterogeneous and the infection rate among randomly chosen sex partners is much higher than among the population as a whole, as partners are selected proportional to their rate of partner change. To calculate male and female equilibrium ("static") HIV prevalence under heterogeneity in scaled mixing rates, for this random mixing model, we proceed along the lines suggested by Dushoff[27], and extended the analysis to a two gender population. In equilibrium, each subgroup with scaled mixing rate x remains constant in size, i.e. each individual HIV death in a group is replaced by exactly one new infected individual. The number of susceptibles in this group is N^m^(x)-I^m^(x). The probability that any of their sex partners is infected is Λ^f^, and the rate at which they seroconvert is xΛ^f^/D^m ^year. Of those infected, a fraction I^m^(x)/D^m ^dies. Thus, the distribution of infected people satisfies, in equilibrium,

I^m^(x) = xΛ^f^(N^m^(x)-I^m^(x))

Using this notation we find that

R_0 _^2 ^= N^m ^_2_N^f ^_2_/N^m ^_1_N^f ^_1_,

and

Im(x)=xΛfNm(x)1+xΛf,
 MathType@MTEF@5@5@+=feaafiart1ev1aaatCvAUfKttLearuWrP9MDH5MBPbIqV92AaeXatLxBI9gBaebbnrfifHhDYfgasaacH8akY=wiFfYdH8Gipec8Eeeu0xXdbba9frFj0=OqFfea0dXdd9vqai=hGuQ8kuc9pgc9s8qqaq=dirpe0xb9q8qiLsFr0=vr0=vr0dc8meaabaqaciaacaGaaeqabaqabeGadaaakeaacqWGjbqsdaahaaWcbeqaaiabd2gaTbaakiabcIcaOiabdIha4jabcMcaPiabg2da9maalaaabaGaemiEaGNaeu4MdW0aaWbaaSqabeaacqWGMbGzaaGccqWGobGtdaahaaWcbeqaaiabd2gaTbaakiabcIcaOiabdIha4jabcMcaPaqaaiabigdaXiabgUcaRiabdIha4jabfU5amnaaCaaaleqabaGaemOzaygaaaaakiabcYcaSaaa@4532@

and

Λm=∫0∞xIm(x)dx∫0∞xNm(x)dx
MathType@MTEF@5@5@+=feaafiart1ev1aaatCvAUfKttLearuWrP9MDH5MBPbIqV92AaeXatLxBI9gBaebbnrfifHhDYfgasaacH8akY=wiFfYdH8Gipec8Eeeu0xXdbba9frFj0=OqFfea0dXdd9vqai=hGuQ8kuc9pgc9s8qqaq=dirpe0xb9q8qiLsFr0=vr0=vr0dc8meaabaqaciaacaGaaeqabaqabeGadaaakeaacqqHBoatdaahaaWcbeqaaiabd2gaTbaakiabg2da9maalaaabaWaa8qCaeaacqWG4baEcqWGjbqsdaahaaWcbeqaaiabd2gaTbaakiabcIcaOiabdIha4jabcMcaPiabdsgaKjabdIha4bWcbaGaeGimaadabaGaeyOhIukaniabgUIiYdaakeaadaWdXbqaaiabdIha4jabd6eaonaaCaaaleqabaGaemyBa0gaaOGaeiikaGIaemiEaGNaeiykaKIaemizaqMaemiEaGhaleaacqaIWaamaeaacqGHEisPa0Gaey4kIipaaaaaaa@4EBB@

Similar relations hold for I^f^(x) and Λ^f^.

These equations, which appear analytically intractable for many scaled mixing rate distributions N^m^(x) and N^f^(x), can be solved iteratively as follows. First a value Λ^f ^is assumed and the equation for I^m^(x) is solved and Λ^m ^calculated. This value is then used to solve the equation for I^m^(x), and this process is continued until convergence is reached. We assume that N^m^(x) ~Gamma(x, p^m^, α^m^), and similarly N^f^(x) ~Gamma(x, p^f^, α^f^), because of the long tailed distribution of the gamma function, reflecting known characteristics of the distribution of rates of partner change[28]. We used Mathematica^® ^to do the calculations[29]. Note that this approach solves the problem of finding a stationary prevalence distribution, not just an overall stationary prevalence, as was done by Williams *et al *[21]. As male-to-female transmission is generally believed to be more efficient than female-to-male transmission, N^f ^_1 _> N^m ^_1_, or p^f^/α^f ^> p^m^/α^m^. This probably also holds in places where MC is rare, such as Nyanza province, in view of the higher female than male HIV prevalences in this province[30]. Interestingly, a recent study on HIV discordant monogamous couples did not find such a difference[31], but such couples may not be representative in terms of important cofactors, for the discordant relationships where most HIV transmission take place. Similarly, as female sex workers can have many more sex partners than any individual heterosexual male, the coefficient of variation in rates of partner change among women is generally believed to be larger than among men, giving (1 +1/p^f^) > (1+1/p^m^). As the coefficient of variation, 1/vp is probably somewhere between 1 and 3, the value of p must be approximately between 0.1 and 1[32]. The effects of a transition from 0% MC to 100% MC on the equilibrium distribution of HIV can be studied by solving the same equations, but assuming N^m^(x) ~Gamma(x, rp^m^, α^m^), where r denotes the relative risk of circumcised men for acquiring HIV infection.

### Compartmental model

As some of the assumptions of the above model are questionable (such as random mixing, a constant rate of change of partnership for each individual, and a fixed transmission probability per partnership), we also explored the effects of male circumcision using a compartmental model [33], originally developed to explore the effects of an HIV vaccine and other interventions on the HIV epidemic[22,34-36]. An additional advantage of this approach is that it can also be used to show the expected time of transition between the current HIV prevalence and the establishment of a new lower equilibrium prevalence.

#### Model structure

**Figure 1:**
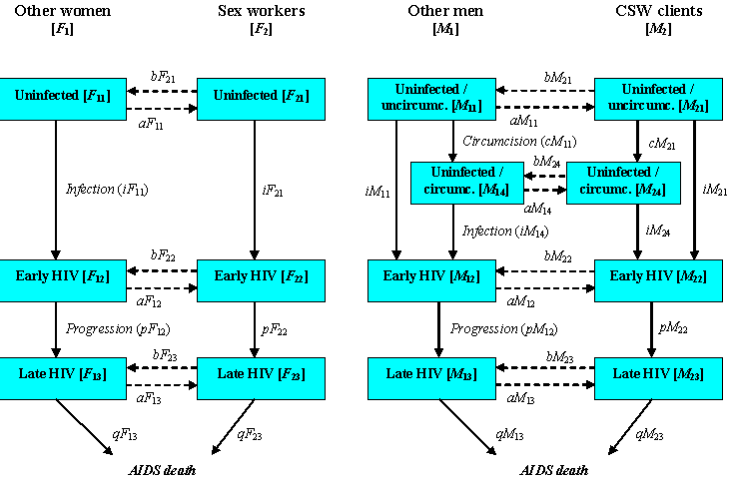
Structure of the compartmental model. Boxes represent compartments, i.e. the states males or females can be in. Arrows represent flows of individuals between compartments. High risk groups are male clients of sex workers, and female sex workers (csw). Disease progression is subdivided into 2 stages: early and late, including AIDS. Individuals (men) move to the circumcised boxes after circumcision. The flow diagram for women is similar except that there exists no circumcised compartment. Symbols refer to compartments and flows formally defined in the Additional file 1 (Compartments: M = male, F = female; first subscript: 1 = low risk group, 2 = high risk group; second subscript: 1 = uninfected, 2 = early HIV, 3 = late HIV, 4 =circumcised and uninfected. Flows: a = from low risk to high risk group, b = from high risk to low risk group, c = circumcision, i = infection, p = progression (to late stage HIV infection), q = death)

This compartmental model does not make the assumption of random mixing, but subdivides the population according to different dimensions (gender, involvement in commercial sex, circumcision status, HIV infection status and stage of infection), into discrete high-risk (male clients and female sex workers for the high-risk groups) and low-risk (general population, not involved in paid sex) groups. Each of these groups is again subdivided into compartments of individuals who are either HIV -infected or not, and, for males, whether they are circumcised or not. In addition, HIV infection is split into two compartments, early and late, to incorporate non-exponential progression to AIDS and mortality. Altogether 6 compartments for (living) women and 8 for (living) men are distinguished. A graph of the model structure is shown in Figure 1.

For women these 6 compartments are:

1. F_11_. HIV negative low risk women, not involved in sex work.

2. F_12_. Women not involved in sex work, during the early stages of HIV infection.

3. F_13_. Women not involved in sex work, during late(r) stage HIV infection.

4. F_21_. HIV negative female sex workers.

5. F_22_. Female sex workers during the early stages of HIV infection.

6. F_23_. Female sex workers during late(r) stage HIV infection.

For men these 8 compartments are:

1. M_11_. Men who are not sex worker clients, HIV uninfected, uncircumcised.

2. M_14_. Men who are not sex worker clients, HIV uninfected, circumcised.

3. M_12_. Men who are not sex worker clients, during the early stages of HIV infection, either circumcised or not.

4. M_13_. Men who are not sex worker clients, during late(r) stage HIV infection, either circumcised or not.

5. M_21_. Male sex worker clients, HIV uninfected, uncircumcised.

6. M_24_. Male sex worker clients, HIV uninfected, circumcised.

7. M_22_. Male sex worker clients, during the early stages of HIV infection, either circumcised or not.

8. M_23_. Male sex worker clients, during late(r) stage HIV infection, either circumcised or not.

No age-structure is incorporated, essentially because reliable quantitative information on age-specific assortative mixing in Africa is not available. However, there is strong empirical evidence that age-mixing in Sub-Saharan Africa is substantial and the HIV epidemic sufficiently "leaky" to expect that ignoring age-specific assortative mixing will not grossly distort what might be expected in reality [37,38]. Interactions, i.e. sexual relationships with the opposite sex, depend on the compartment (high-risk or low-risk) that individuals are in. For example, "non-client" men do not visit sex workers at all. HIV transmission between low risk population groups consists of two types, namely through entering into a new marriage-like or other stable relationship (marriages, regular partners, not necessarily monogamous), or through "leakage" from infected individuals, which involves both all non-paid casual sex and all existing sexual relationships at the time an individual becomes HIV infected. The risk of transmission to initially HIV negative partners in new marriage-like relationships is assumed to be determined by the product of the parameters *marrate_male *("marriage-rate" of men) and *stabfactor *(a multiple of the per-contact transmission rate with a sex worker). Instead of making this product one parameter, we used two parameters in order to make sure that the number of such stable relationships is the same for men and for women. This was achieved by making the annual rate of entering into a new stable relationship a (fixed) parameter for men, but "demand driven" for women. The annual transmission through "leakage" is set by an independent parameter (*leak*) that sets the level of leakage when all partners would be initially HIV uninfected. However, the amount of leakage decreases when HIV prevalence increases due to "pre-emption" (i.e. some partners cannot be infected as they are already HIV seropositive). The relative risk of male-to-female leakage and female-to-male leakage is assumed to be the same as that for male-to-female and female-to-male transmission probability per high risk (FSW-client) contact. The only mode of HIV transmission is assumed to be heterosexual. The co-factor effects of sexually transmitted infections are not explicitly modelled, but are assumed to affect the average risk of sexual transmission, as they did in the studies on which transmission parameter values were based. Demand for sex work, i.e. the rate of becoming a sex-worker client, and the frequency of intercourse with sex-workers is assumed to be an independent mechanism, but the rate of becoming a sex worker and also the average number of client contacts per sex worker are demand driven. Specifically, the rate of becoming a sex worker is assumed to depend exponentially on the per sex worker annual number of contacts with clients. Since group membership (e.g. being a sex worker) of individuals is not fixed, but may change over time (e.g. women may practice sex work for a few years only), individuals may move between high risk and low risk compartments. Transitions between compartments (groups) are given by differential equations, which are solved numerically using ModelMaker^® ^(version 3)[39]. The model compartments, flows, variables and parameters are formally described in the Additional file 1.

### Choice of parameter values

We adjusted the parameters of the model to incorporate recent (higher) estimates of female-to-male HIV transmission risk (*fm_risk*) per high-risk contact among uncircumcised high-risk men[40], and to obtain a reasonable fit to prevalence estimates in two regions in Africa where MC is uncommon (approximately 10% of all adult males) and HIV prevalence is high,*viz*. Nyanza Province in Kenya, and Botswana in southern Africa. Recent HIV prevalence estimates[1] of Botswana for the entire 15–49 years old population are somewhat lower than earlier ones based on ANC surveillance which we used for our study so that our model perhaps does not adequately reflect the whole of Botswana and may at best represent the worst affected areas of this country. The major difference between the two sites was that the number of non-commercial casual contacts was assumed to be higher in the "Botswana" scenario than in the "Nyanza" scenario, by choosing a higher value of *leak*. Other parameters were either chosen on the basis of published data or plausible information, or were "tweaked" (tuned) to provide the desired baseline prevalences[35].

Specifically, the parameters *cust *and *uncust *representing the annual rates by which men become clients of sex workers and the rate of moving back to low risk were based on "estimates" that approximately 20% of men are clients of sex workers at any given time[41,42]. The parameters controlling the rates of becoming a sex-worker and moving back to the low risk groups were based on the assumption that slightly less than 10% of women at any time exchange sex for money[43], in combination with the assumption that women tend to remain sex-worker for only a few years. The contact rate between clients and sex workers of once every two weeks was suggested *inter alia *by a report of 11 such contacts during 12 man-weeks in a probably biased sample of male clients in Uganda[44], and a study reporting clients visiting sex-workers in Nyanza, Kenya once or twice weekly [45]. As the latter study also reported that most clients were not consistently using condoms with sex workers we chose the baseline level of unprotected sex *unprot *due to no condom use or incorrect use to be 80%. The parameters *hivprog*, and *mu_aids *were based on the assumption of an 8 years duration from infection to AIDS death and an arbitrary 50:50 division into early and a late stage infection. Parameters *mu_pos *and *mu_neg *of 0.028 and 0.026 respectively, were chosen on the basis of a duration of 35–40 years between entering the sexually active population and leaving this population due to ageing or death and the assumption of marginally higher mortality rates among early HIV positive individuals. The parameters *malegr *and *femgr *of 0.04 were chosen to obtain realistic population growth rates. These parameters determine the influx into the male (at annual rate malegr* population) and female (at annual rate femgr* population) adult sexually active population due to the ageing of children. The ratio of male-to-female (*mf_risk*) and female-to-male (*fm_risk*) risk was chosen on the basis of observed prevalence ratios. The parameters *marrate_male*, *stabfactor*, and *leak *were tuned to yield desired baseline prevalences. However, it may be noteworthy that there is some arbitrariness in the choice of these parameter values, as it appeared that the two non sex-worker transmission mechanisms were largely interchangeable.

On the basis of the Orange farm trial, we assumed a relative risk of MC for female-to-male transmission of 0.4. We further assumed absence of behavioural disinhibition (risk compensation, i.e. increased risk taking) after circumcision, and no effect of male circumcision on male-to-female transmission

In our model specification, men were not circumcised instantaneously, but uncircumcised men were recruited as a constant rate leading to a specified (50% or 80%) equilibrium prevalence of circumcision among HIV uninfected men. As initially most men are uncircumcised, the number of men getting circumcised is higher initially than in equilibrium when circumcision numbers are just high enough to maintain equilibrium circumcision levels.

#### Sensitivity analyses

For our main (baseline) model specifications, we assumed a relative risk of MC for female- to-male transmission of 0.4, no behavioural disinhibition (risk compensation), and no effect on male-to-female transmission. These three assumptions were subjected to sensitivity analyses as follows:

1) As a first sensitivity analysis we considered two "extreme" relative risks of the effect of circumcision on female-to-male transmission, *viz*. a RR of 0.25, and a RR of 0.6 for female-to-male transmission. These values correspond roughly to the per-protocol estimate of the RR from the Orange Farm trial, and the upper limit of the 90% CI of the RR, respectively, and thereby constitute moderately optimistic and pessimistic estimates. The high RR value of 0.6 also somewhat exceeds the estimated effects in Uganda and Kenya and thus seems to be a "safe" upper limit of the RR.

2) As a second sensitivity analysis we explored the effect of a (moderate) protective effect of MC on male-to-female transmission, which we had assumed absent in our main analysis. Instead of a relative risk of 1 for MC (i.e. no effect on male-to-female transmission), we assumed a relative risk of 0.75.

3) As indicated above, the unadjusted and adjusted (for behavioural variables) protection levels (i.e. 1-RR) estimates obtained from the Orange Farm trial were very close (0.60 and 0.61 respectively). It thus appears that in this trial, negative behaviour change (disinhibition or risk compensation) following circumcision, leading to higher risk taking, was not an important effect. However, this may be partly due to the intensive counselling that trial subjects received, and in practice, after program scale-up, some degree of disinhibition would seem plausible. As a third sensitivity analysis, we therefore considered the possibility that circumcised men would abandon condom use in high-risk sexual contacts (assumed to be 20% effectively initially).

4) As a fourth sensitivity analysis, we explored the impact of changing the assumption of uniform infectiousness (with the underlying idea that most infections take place both early ***and ***late during infection), to one in which individuals are more (on average 5 times more) infectious during the first year after infection than during the remainder of their infection. This should also provide a reasonable approximation to the situation in which infectiousness is extremely high for only a few weeks, either due to high viraemia during primary infection [46] or due to the effect of a concomitant sexually transmitted infection that increases both HIV susceptibility and infectiousness.

5) As a fifth and final sensitivity analysis, we explored several different other "realistic" mixes of sexual behaviour parameters (client-CSW contacts, other casual contacts, and new stable partnerships) that were consistent with the same assumed male and female baseline HIV prevalences.

## Results

### Random mixing model

Three scenarios with different degrees of heterogeneity in rates of partner change were considered. In all three scenarios, it was assumed that per-partnership male-to-female transmission was 2.5 times more efficient than female-to-(uncircumcised) male transmission, and that MC has a 60% protective effect.

**Figure 2:**
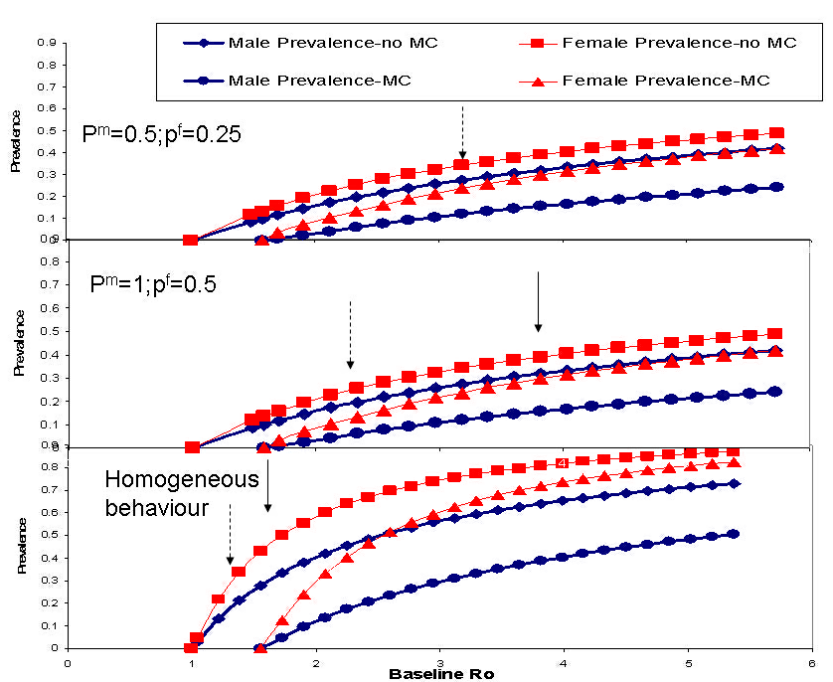
The relationship between R_0_, male circumcision (MC), and sex-specific equilibrium HIV prevalence, under a random mixing assumption, assuming different levels of heterogeneity of sexual behaviour (rates of partner change). The distribution of male and female rates of partner change (x) are assumed to be N^m^(x) ~Gamma(x, p^m^, α^m^) and N^f^(x) ~Gamma(x, p^f^, α^f^) respectively. Bottom panel: homogeneous rates of partner change; middle panel: moderate heterogeneity in rates of partner change with parameters p^m ^= 1 & p^f ^= 0.5; top panel: high heterogeneity in rates of partner change with parameters p^m ^= 0.5 & p^f ^= 0.25. Continuous arrows indicate the approximate position of "Botswana" and the dotted arrow indicates the approximate position of "Nyanza".

Figure 2, bottom panel, shows the effect of MC on the equilibrium HIV prevalence when all individuals have identical contact rates, i.e. a homogeneous population. HIV prevalence levels vary considerably with the basic reproductive rate. In populations where male circumcision is not practised, HIV prevalence is zero when R_0 _≤ 1, and rises to about 85% among women and 60% among men at levels of R_0 _of 4–5. In populations where male circumcision is practised, HIV prevalence is zero when (the non-MC) R_0 _equals 1.5, and rises to about 80% among women and 45% among men at levels of (non-MC) R_0 _of 4–5. For realistic levels of HIV prevalence, R_0 _would be between 1 and 2. For example, for "Botswana", R_0 _would only be about 1.5, making it possible to completely eliminate the infection from the population.

Figure 2, middle panel, shows the effect of male circumcision on the equilibrium male and female HIV prevalences, when the parameters for heterogeneity, p^m ^and p^f^, are set to 1 and 0.5. In populations where male circumcision is not practised, HIV prevalence is about 40% for females and 30% for males (similar to "Botswana"), at an R_0 _of 4. MC would reduce this prevalence to about 25–30% for females and 15% for males.

Figure 2, top panel, shows these curves for values of p^m ^and p^f ^set to 0.5 and 0.25. In populations where male circumcision is not practised, HIV equilibrium prevalence is about 30% for females and 25% for males at levels of R_0 _of 4–5. MC would reduce this prevalence to about 25% for females and 12% for males. The value of R_0 _in "Botswana" would exceed 6, making it extremely difficult to eliminate the infection from the population.

### Compartmental Model

**Figure 3:**
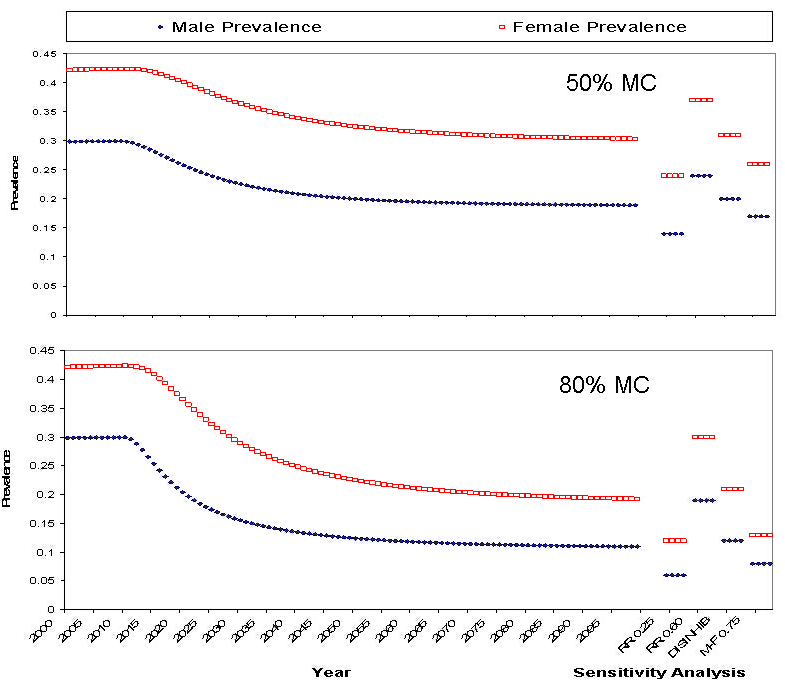
The impact of male circumcision (MC) on HIV prevalence in the Botswana setting, according to our compartmental model, with high, 80%, MC uptake (bottom panel) and moderate, 50%, MC uptake (top panel). Predictions are for the period 2000–2100, when male circumcision is introduced in 2010. In addition the figure shows the results of 4 different sensitivity analyses: RR0.25: if protection afforded by circumcision would be as high 75% (RR 0.25); RR0.60: if it would be as low as 40% (RR 0.60); DISINHIB: if it would lead to disinhibition in the sense that condom use in high risk sex would be abandoned; MF 0.75: if male circumcision would reduce the risk of male-to-female transmission by 25%.

**Figure 4:**
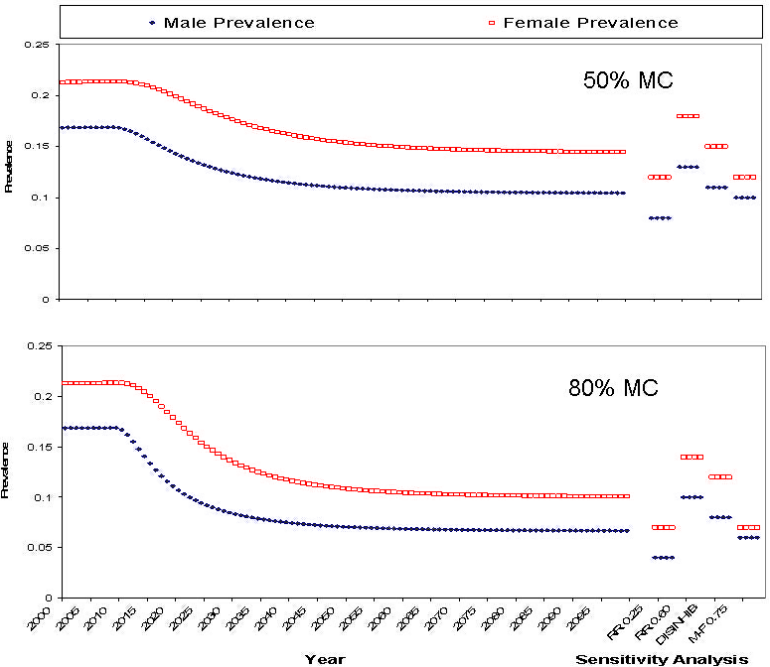
The impact of male circumcision (MC) on HIV prevalence in the Nyanza setting, according to our compartmental model, with high, 80%, MC uptake (bottom panel) and moderate, 50%, MC uptake (top panel). Predictions are for the period 2000–2100, when male circumcision is introduced in 2010. In addition the figure shows the results of 4 different sensitivity analyses:RR0.25: if protection afforded by circumcision would be as high 75% (RR 0.25); RR0.60: if it would be as low as 40% (RR 0.60); DISINHIB: if it would lead to disinhibition in the sense that condom use in high risk sex would be abandoned; MF 0.75: if male circumcision would reduce the risk of male-to-female transmission by 25%.

Figures 3 and 4 show the relationship between HIV prevalence and calendar time. The model was run for the period 1950–2100. Male circumcision was introduced in the year 2010. The first 50 years (1950–1999) were used as a "burn in", using all of the demographic and transmission parameters (but without MC) described above, in order to reach approximate pre-circumcision equilibrium HIV prevalence levels associated with these parameters. Note that this is not assumed to adequately represent the pre-2000 HIV epidemic; if only because HIV was probably introduced some time during the 1970s. After introduction of MC, it takes several decades to reach the new equilibrium associated with the lower transmission risks. It even takes somewhat longer than a decade to reach half the ultimate impact. In part this is because prevalent cases are removed only slowly by mortality. However, part of this is also due to the assumption that not all men are circumcised instantaneously, but rather, more realistically, that uncircumcised men are recruited at a fixed rate, and the pool of uncircumcised men diminishes only gradually.

Figures 3, 4 also show the equilibrium (i.e. in the year 2100) male and female HIV prevalence under the first three sensitivity analysis assumptions.

#### High (80%) MC uptake

Figure 3, lower panel, shows the effects of the introduction of MC in "Botswana" for a program that manages to increase MC from 10% to 80% of susceptible (HIV negative) males over a period of about 10 years. Female HIV prevalence decreased over time from slightly over 40% to about 20%. Male HIV prevalence decreased from about 30% to slightly over 10%. Figure 4, lower panel, shows the same effects for "Nyanza Province" with 80% MC uptake over 10 years. Female HIV prevalence decreased over time from 22% to about 10%. Male HIV prevalence decreased from about 17% to 7%.

#### Low (50%) MC uptake

Figure 3, top panel, shows the effects of MC in "Botswana" for a program that achieves a male circumcision rate of only 50%, also over about a 10 year period. Female HIV prevalence decreased over time from slightly over 40% to about 30% Male HIV prevalence decreased from about 30% to 20%. Figure 4, top panel, shows the same scenario as Figure 3, top panel, but for "Nyanza Province". Female HIV prevalence decreased over time from 22% to about 15%. Male HIV prevalence decreased from about 17% to slightly over 10%.

#### Sensitivity Analyses

The first three sensitivity analyses clearly show (Figures 3, 4) that the impact of MC is very sensitive to assumptions about the degree of protection (relative risk) afforded by male circumcision, especially in terms of female-to-male transmission. However, if MC also reduces the efficiency of male-to-female HIV transmission, then there could be a substantial further impact on equilibrium HIV prevalence, among both men and women. Behavioural disinhibition (risk compensation) had a much smaller impact, perhaps because the potential for disinhibition was assumed to be small (reduction of condom use in contacts with sex workers from 20% to 0%; no effect on transmission in other partnerships).

The fourth sensitivity analysis (non-homogeneous infectiousness) did not substantially affect the ultimate equilibrium HIV prevalence (for the same pre-intervention equilibrium levels), but somewhat accelerated the rate of approximation to the new equilibrium, especially during the early phase of the introduction of the intervention. All parameter combinations tried under the fifth sensitivity analysis (varying sexual mixing parameters) yielded very similar effects of MC on HIV prevalence over time (not shown).

## Discussion

There are different approaches to estimating the potential impact of male circumcision on the HIV epidemic. As noted above, the simplest one compares regions in Africa where MC is universally practised with regions where it is exceptional. This approach has the drawback that these regions were not randomly allocated to MC or not, and that confounding is a real possibility. In addition, both within MC areas and within non-MC areas, there are substantial heterogeneities in HIV prevalence which are not well understood[47]. An advantage of this ecological approach, however, is that it does not depend on estimates of the effect of MC, and it automatically includes any effect of MC not taken into account in the model, such as a possible higher protection afforded by MC for the most sexually active men, or an effect on male-to-female transmission.

The other approach to exploring the effects of MC is mathematical modelling. While the techniques for modelling complex epidemics of infectious diseases are well developed, modelling the HIV epidemic is plagued by substantial uncertainty around the estimates for most key parameters, such as per-contact or per-partnership transmission risks, or the effects of co-factors on these risks. In addition, sexual networks in Africa, as elsewhere, are poorly understood, and the methodology for studying them, such as sexual behaviour surveys, is still unreliable[48,49]. Fitting models to observed data to estimate parameters is not an entirely satisfactory solution, as the number of unknown parameters involved in any realistic model usually exceeds the available data, which also tend to be selective and scarce. Even the actual HIV prevalence in many countries, including Botswana, is a moot issue[50]. The amount of heterogeneity in sexual contact rates is an essential parameter governing the effect of MC for given equilibrium HIV prevalences. For low rates of heterogeneity, prevalence rates as commonly observed in Africa would correspond to rather low basic reproductive numbers, R_0_, and in such contexts, MC could well be able to eliminate HIV altogether. Clearly, however, this would be unrealistic, as sexual behaviour is highly heterogeneous, and we know that some countries where MC is practised universally still have significant HIV transmission, albeit not as great as in countries where MC is rare. Values of R_0 _in Africa are not well known. They can be estimated from the intrinsic doubling time of the epidemic, e.g. slightly over 1 year in South Africa[51], giving rise to a very high R_0 _estimate of approximately 7. However, this estimate is based on the assumption that the number of secondary infections generated by an individual infected with HIV would be constant over the whole period between infection and death. As most secondary infections are probably generated during the early phases of infection, the true value of R_0 _should be much lower.

To partly overcome model uncertainties, and to explore the sensitivity of findings to specific model choices (model sensitivity), we attempted to estimate and compare the impact of MC on the HIV epidemic using two different modelling approaches, and to identify where predictions agree and where they do not. Both approaches indicate a substantial impact of MC on both male and, to a lesser extent, female HIV prevalence. The random mixing model, when assuming a gamma distributed rate of partner change, suggested decreases in male prevalence of at least 40%, but for realistic prevalences perhaps appreciably higher, with approximately a 60% reduction in prevalence among men and 30% among women. These estimates are similar to those predicted using our compartmental model, although the assumptions for these models were different, with an increase in MC rates from 10% to 50% or 80% in the latter model as opposed to from 0 to 100% in the former. This similarity suggests that our compartmental model in which individuals can move between high-risk and low risk compartments at significant rates can be closely approximated by a random mixing model. Our results also agree with those of Williams *et al *[21], although we predict a somewhat stronger effect of MC than they do. For example, for Botswana, they predict a reduction in incidence of 1.17%/year. Taking into account the average duration of HIV disease, between HIV infection and death, this corresponds to a decrease in prevalence of approximately 10% from 37.3% to approximately 27%, i.e. a relative reduction of 25–30%. The somewhat stronger effect predicted from our models appears to be due to a different approach to handling heterogeneity in sexual behaviour.

Results from our compartmental model indicate that, although the declines in HIV prevalence after male circumcision programs are expanded are substantial, it may take some time to reach the new equilibrium prevalences, particularly as it may take time to reach high coverage with male circumcision services. Even with a vigorous program, it may take as much as two decades to reach equilibrium associated with the protection afforded by MC. Nevertheless, within a few years, a decline in prevalence should set in and the burden of HIV should diminish rapidly, with the incidence naturally declining faster than the prevalence. An interesting finding is the generally concave shape of the relationship between R_0 _and prevalence. The implication of this shape is that MC may have a positive synergy with other interventions that reduce R_0 _by a fixed amount. Interventions that reduce R_0 _by a fixed percentage (perhaps a more probable scenario) do not have this advantage. Plots (not shown) of the prevalence, as predicted by the random mixing model, against the logarithm of R_0_, show approximately linear relationships, suggesting that the absolute decline in prevalence contributed by additional interventions would be approximately constant. Of course, the *relative *decline of additional interventions would increase after the introduction of MC. Thus, MC would likely have positive synergistic interactions with other interventions, such as sex worker preventive interventions.

Our findings appear to be relatively insensitive to behavioural disinhibition (risk compensation), perhaps because it was assumed that the potential for disinhibition was small, given that most sex worker clients (at least in Nyanza province [45]) do not use condoms consistently in the baseline scenario. Naturally, our results were quite sensitive to the assumed level of protection (relative risk) afforded by male circumcision, both on female-to-male and male-to-female transmission. As ongoing MC trials have been stopped and other trials are unlikely to be carried out in the future, estimates of the protection afforded by MC are likely to remain somewhat imprecise.

As our models lack an age structure, one important aspect of a general MC intervention that is not addressed by our models is the effect of the intervention on the average age of infection, and consequently the development of AIDS and death. When the force-of-infection declines, the average age of infection increases, and consequently the number of life-years lost, for every incident HIV infection, also decreases. Precise estimation of this effect would require detailed knowledge of age-specific sexual behaviour patterns. However, it is clear that in terms of the number of life-years lost, the effect of MC on the HIV epidemic may be larger than that measured solely in terms of HIV prevalence, and the cost-effectiveness of MC interventions would thus be enhanced. Rough estimates of the gains in life expectancy that large scale MC programs may yield can be gleaned from estimates of decreases in life-expectancy attributable to AIDS[26]. For example, in Zimbabwe, it is estimated that AIDS has reduced life expectancy by 22.2 years. Let us assume that, in equilibrium, approximately 2%, i.e. roughly half the gross birth rate (as only males will be circumcised) in many parts of Africa, will be eligible for circumcision, at a cost of US$20 – US$40 per procedure, depending on country, method of implementation, etc. Then the per capita cost of maintaining a circumcision programme will be approximately 2% of the cost of a male circumcision, i.e. US$ 0.40 – US$ 0.80. The benefits of such a MC program would be a reduction in the loss in life expectancy due to AIDS by 20–50%; i.e., regaining 5–11 years of life expectancy. Alternatively, it amounts to reducing an incidence rate of approximately 1% per capita (the whole population, not only adults) by 20–50%. At less than a dollar per capita annually, this would translate into a cost of approximately 100–500 US$ per HIV infection averted. Similar cost estimates ($117-$306) were calculated by Kahn *et al *for Gauteng, South Africa[52]. This would make the cost-effectiveness of MC comparable to or better than other accepted cost-effective interventions, such as anti-retroviral treatment for prevention of mother-to-child HIV transmission[53].

We have not addressed the issue of optimal targeting of MC programs, but rather assumed either universal circumcision or random uptake of circumcision. It may be most efficient to focus circumcision services on high-risk men, but these men may not be easy to identify. Alternatively, circumcising successive cohorts of young men just prior to onset of sexual debut (e.g. 12–16 year-olds) may be highly efficient. Fortunately, this is the age range of most circumcisions occurring traditionally in eastern and southern Africa. Actual MC service delivery programs will likely depend more on factors such as cultural preferences and country-specific infrastructure than on mathematically optimal service and resource allocation. Similarly, negative effects of large scale circumcision such as adverse effects of surgery, will also depend on specific delivery programs. These should be assessed, preferably in realistic pilot projects, and taken into account in program specific effectiveness and cost-effectiveness analyses, before trial projects are scaled-up.

## Conclusion

Large-scale uptake of male circumcision services in African countries with high HIV prevalence and where male circumcision is not now routinely practised, could lead to substantial reductions in HIV transmission and prevalence over time among both men and women. The full impact of MC on HIV-related illness and death will only be apparent after more than a decade. Our findings support qualitatively similar findings by other authors using different models [21]. Together with those findings, our results indicate the approximate range of reduction in HIV prevalence that would result from large scale introduction of male circumcision programmes in Africa.

## Competing interests

Stephen Moses and Robert Bailey are primary investigators in a randomized clinical trial examining the impact of male circumcision on HIV incidence in Kenya, funded by the United States National Institutes of Health (NIH) and the Canadian Institutes of Health Research (CIHR).

## Authors' contributions

All authors contributed to the design of the study and the writing of the paper. NN and SdV developed and ran the compartmental model. NN also analyzed the random mixing model. All authors read and approved the final manuscript.

## Pre-publication history

The pre-publication history for this paper can be accessed here:



## Supplementary Material

Additional File 1 Formal Structure of the Compartmental Model. The file describes the formal mathematical structure of the compartmental model, and specifies parameter values used. Click here for file
