# Supplementary material for: Modelling the public health impact of male circumcision for HIV prevention in high prevalence areas in Africa
Source: BMC Infect Dis. 2007 Mar 13;7:16. doi: 10.1186/1471-2334-7-16 (PMC1832203; doi:10.1186/1471-2334-7-16)
Supplement: Additional File 1 — Formal Structure of the Compartmental Model. The file describes the formal mathematical structure of the compartmental model, and specifies parameter values used. [file 1471-2334-7-16-S1.doc]

**Formal structure of the compartmental model**

**Note: parameters names are in *italics,* variable names are in normal font.**

**Names of compartments and flows were chosen as follows. Compartments: M = male, F = female; first subscript: 1 = low risk group, 2 = high risk group; second subscript: 1 = uninfected, 2 = early HIV, 3 = late HIV, 4 =circumcised and uninfected. Flows: a = from low risk to high risk group, b = from high risk to low risk group, c = circumcision, i = infection, p = progression (to late stage HIV infection), q = death.**

COMPARTMENTS

| **Symbol**  **Figure 1** | **Equation** |
| --- | --- |
| F_11_ | -*mu_neg**F_11_ - aF_11_ + bF_21_ - iF_11_ + population**femgr* |
| F_12_ | -*mu_pos**F_12_ - aF_12_ + bF_22_ + iF_11_ - pF_12_ |
| F_13_ | **-***mu_pos******F_13_ - aF_13_ + bF_23_ + pF_12_ - qF_13_ |
| F_21_ | **-***mu_neg******F_21_ + aF_11_ - bF_21_ - iF_21_ |
| F_22_ | -*mu_pos**F_22_ + aF_12_ - bF_22_ + iF_21_ - pF_22_ |
| F_23_ | -*mu_pos**F_23_ + aF_13_ - bF_23_ + pF_22_ - qF_23_ |
| M_11_ | -*mu_neg**M_11_ - aM_11_ + bM_21_- iM_11_ - cM_11_ + population**malegr* |
| M_12_ | *-mu_pos**M_12_ - aM_12_ + bM_22_ + iM_11_ - pM_12_ + iM_14_ |
| M_13_ | **-***mu_pos******M_13_ **-** aM_13_ + bM_23_ + pM_12_ - qM_13_ |
| M_14_ | -*mu_neg**M_14_ - aM_14_ + bM_24_ + cM_11_ - iM_14_ |
| M_21_ | -*mu_neg**M_21_ + aM_11_ - bM_21_ - iM_21_ - cM_21_ |
| M_22_ | -*mu_pos**M_22_ + aM_12_ - bM_22_ + iM_21_ - pM_22_ + iM_24_ |
| M_23_ | **-***mu_pos******M_23_ **+** aM_13_ - bM_23_ + pM_22_ - qM_23_ |
| M_24_ | -*mu_neg**M_24_ + aM_14_ - bM_24_ + cM_21_ - iM_24_ |
| AIDSdeath_f (female) | qF_13_ + qF_23_ |
| AIDSdeath_m (male) | qM_13_ + qM_23_ |

FLOWS (transitions between compartments)

| **Symbol**  **Fig.1** | **From** | **To** | **Equation** |
| --- | --- | --- | --- |
| aF_11_ | F_11_ | F_21_ | *prof* * F_11_* exp (annualCSWcontacts / *mkt* - 1) |
| aF_12_ | F_12_ | F_22_ | *prof* * F_12_* exp (annualCSWcontacts / *mkt* - 1) |
| aF_13_ | F_13_ | F_23_ | *prof* * F_13_* exp (annualCSWcontacts / *mkt* - 1) |
| aM_11_ | M_11_ | M_21_ | *cust* * M_11_ |
| aM_12_ | M_12_ | M_22_ | *cust* * M_12_ |
| aM_13_ | M_13_ | M_23_ | *cust* * M_13_ |
| aM_14_ | M_14_ | M_24_ | *cust* * M_14_ |
| bF_21_ | F_21_ | F_11_ | *unprof* * F_21_ |
| bF_22_ | F_22_ | F_12_ | *unprof* * F_22_ |
| bF_23_ | F_23_ | F_13_ | *unprof* * F_23_ |
| bM_21_ | M_21_ | M_11_ | *uncust* * M_21_ |
| bM_22_ | M_22_ | M_12_ | *uncust* * M_22_ |
| bM_23_ | M_23_ | M_13_ | *uncust* * M_23_ |
| bM_24_ | M_24_ | M_14_ | *uncust* * M_24_ |
| cM_11_ | M_11_ | M_14_ | mc_rate_men * M_11_ |
| cM_21_ | M_21_ | M_24_ | mc_rate_clients * M_21_ |
| iF_11_ | F_11_ | F_12_ | leakmen * (M_12_ + M_13_ + M_22_ + M_23_) * F_11_ / non_csw +  F_11_ * *stabfactor* * *mf_risk* * marrate_female * (M_12_ + M_13_) / non_clients |
| iF_21_ | F_21_ | F_22_ | F_21_ * annualCSWcontacts * *mf_risk* * *unprot*  * (M_22_ + M_23_) / clients |
| iM_11_ | M_11_ | M_12_ | leakwomen * (F_12_ + F_13_ + F_22_ + F_23_) * M_11_ / non_clients +  M_11_ * *stabfactor* * *fm_risk* * *marrate_male* * (F_12_ + F_13_) / non_csw |
| iM_14_ | M_14_ | M_12_ | *mc_relat_risk** (leakwomen * ( F_12_ + F_13_+ F_22_ + F_23_) * M_14_ / non_clients + M_14_ * *stabfactor* * *fm_risk* * *marrate_male* * (F_12_ + F_13_) / non_csw ) |
| iM_21_ | M_21_ | M_22_ | M_21_ * *cont_rate* * *fm_risk* * *unprot* * (F_22_ + F_23_) / csw |
| iM_24_ | M_24_ | M_22_ | *mc_relat_risk* * (M_24_ * *cont_rate* * *fm_risk* * *unprot* * (F_22_ + F_23_) / csw) |
| pF_12_ | F_12_ | F_13_ | *hivprog* * F_12_ |
| pF_22_ | F_22_ | F_23_ | *hivprog* * F_22_ |
| pM_12_ | M_12_ | M_13_ | *hivprog* * M_12_ |
| pM_22_ | M_22_ | M_23_ | *hivprog* * M_22_ |
| qF_13_ | F_13_ | AIDSdeath_f | *mu_aids* * F_13_ |
| qF_23_ | F_23_ | AIDSdeath_f | *mu_aids* * F_23_ |
| qM_13_ | M_13_ | AIDSdeath_m | *mu_aids* * M_13_ |
| qM_23_ | M_23_ | AIDSdeath_m | *mu_aids* * M_23_ |

VARIABLES

| **Variable Name** | **Defining equation** |
| --- | --- |
| **non_csw** | F_11_ + F_12_ + F_13_ |
| **csw** | F_21_ + F_22_ + F_23_ |
| **females** | non_csw + csw |
| **non_clients** | M_11_ + M_12_ + M_13_ + M_14_ |
| **clients** | M_21_ + M_22_ + M_23_ + M_24_ |
| **males** | non_clients + clients |
| **population** | males + females |
| **female_prevalence** | (females - F_11_ - F_21_) / females |
| **male_prevalence** | (males - M_21_ - M_11_ - M_14_ - M_24_) / males |
| **circumcision_prevalence** | (M_14_ + M_24_) / (M_11_ + M_14_ + M_21_ + M_24_) |
| **annualCSWcontacts** | *cont_rate* * clients / csw |
| **leakwomen** | *leak* * 2 * *fm_risk* / *(fm_risk* + *mf_risk)* |
| **leakmen** | *leak* * 2 * *mf_risk* / *(mf_risk* + *fm_risk)* |
| **mc_rate_men** | *mc_post* for t>60 (i.e. the year 2010); *mc_pre* by default |
| **mc_rate_clients** | *mc_post* for t>60; *mc_pre* by default |
| **marrate_female** | *marrate_male* * non_clients / non_csw |

**Model Parameters (values)**

**Note: values that are identical for all scenarios are only shown once.**

| **Parameter name** | Interpretation | **“Botswana”** | | **“Nyanza”** | |
| --- | --- | --- | --- | --- | --- |
|  | Parameter description/interpretation | **50%** | **80%** | **50%** | **80%** |
| ***femgr*** | Annual growth rate female population | 0.04 | | | |
| ***malegr*** | Annual growth rate male population | 0.04 | | | |
| ***mu_neg*** | Annual mortality rate HIV negatives | 0.026 | | | |
| ***mu_pos*** | Annual mortality rate (non AIDS) HIV positive | 0.028 | | | |
| ***mu_aids*** | Annual rate of AIDS death among late stage HIV infected | 0.25 | | | |
| ***hivprog*** | Annual rate of developing late stage HIV among early stage HIV infected | 0.25 | | | |
| ***fm_risk*** | Probability of female to (uncircumcised) male transmission per high-risk contact. | 0.0135 | | | |
| ***mf_risk*** | Probability of male-to-female transmission per high-risk contact. | 0.03 | | | |
| ***marrate_male*** | Annual rate of establishing stable relationships (men) | 0.232 | | | |
| ***stabfactor*** | Multiplier for stable relationships | 25 | | | |
| ***leak*** | Annual transmission to non-commercial non marital partners | 0.11 | | 0.065 | |
| ***cust*** | Annual rate of becoming CSW client | 0.025 | | | |
| ***uncust*** | Annual rate of becoming low risk among CSW clients | 0.1 | | | |
| ***prof*** | Annual rate of becoming CSW among low risk women when CSW have *mkt* clients annually | 0.025 | | | |
| ***unprof*** | Annual rate of becoming low risk among CSW | 0.25 | | | |
| ***cont_rate*** | Annual number CSW contacts per clients (rate) | 26 | | | |
| ***mkt*** | Parameter controlling the rate of becoming CSW in response to demand | 1000 | | | |
| ***unprot*** | Effective lack of condom use CSW clients | 0.8 | | | |
| ***mc_relat_risk*** | Protection afforded by circumcision | 0.40 | | | |
| ***mc_pre*** | Annual rate of MC before intervention | 0.008 | | | |
| ***mc_post*** | Annual rate of MC after intervention | 0.075 | 0.30 | 0.075 | 0.30 |
